# Type 2 immunity-dependent reduction of segmented filamentous bacteria in mice infected with the helminthic parasite *Nippostrongylus brasiliensis*

**DOI:** 10.1186/s40168-015-0103-8

**Published:** 2015-09-17

**Authors:** W. Florian Fricke, Yang Song, An-Jiang Wang, Allen Smith, Viktoriya Grinchuk, Chenlin Pei, Bing Ma, Nonghua Lu, Joseph F. Urban, Terez Shea-Donohue, Aiping Zhao

**Affiliations:** Department of Microbiology and Immunology, Institute for Genome Sciences, University of Maryland School of Medicine, Baltimore, MD USA; Department of Nutrigenomics, University of Hohenheim, Stuttgart, Germany; Department of Radiation Oncology, University of Maryland School of Medicine, Baltimore, MD USA; U.S. Department of Agriculture, Agriculture Research Service, Beltsville Human Nutrition Research Center, Diet, Genomics, and Immunology Laboratory, Beltsville, MD USA; Department of Gastroenterology and Hepatology, The First Affiliated Hospital of Nanchang University, Nanchang, China

**Keywords:** Helminth parasite, *Nippostrongylus brasiliensis*, Type 2 immunity, IL-13, STAT6, Antimicrobial peptides, Segmented filamentous bacteria, IL-17, Microbiota

## Abstract

**Background:**

Dynamic interactions between the host and gastrointestinal microbiota play an important role for local and systemic immune homeostasis. Helminthic parasites modulate the host immune response, resulting in protection against autoimmune disease but also increased susceptibility to pathogen infection. The underlying mechanisms remain largely unknown.

**Results:**

We showed that the type 2 immune response to enteric *Nippostrongylus brasiliensis* infection in mice was associated with altered intestinal mucin and AMP expression and shifts in microbiota composition. Most strikingly, infection reduced concentrations of intestinal segmented filamentous bacteria (SFB), known inducers of T helper 17 cells, and IL-17-associated gene expression. Infected mice deficient in IL-13 or STAT6 did not reduce SFB or IL-17, and exogenous IL-25 replicated the effects of parasite infection in wild type mice.

**Conclusions:**

Our data show that parasite infection acts through host type 2 immunity to reduce intestinal SFB and expression of IL-17, providing an example of a microbiota-dependent immune modulation by parasites.

**Electronic supplementary material:**

The online version of this article (doi:10.1186/s40168-015-0103-8) contains supplementary material, which is available to authorized users.

## Background

Homeostasis of the mammalian gastrointestinal (GI) tract depends on a complex network of interactions between the host and microbiota, including parasitic nematodes, bacteria, viruses, and others [1]. To describe the potential of individual microbiota members to exert both beneficial and detrimental effects on the host, the term “pathobiont” has been suggested for symbiotic organisms that induce pathology under certain conditions [2]. For example, segmented filamentous bacteria (SFB), Gram-positive members of the *Clostridiaceae* family that colonize various vertebrate species including humans [3, 4], promote specific T helper (Th)17 differentiation through MHCII-dependent antigen presentation by intestinal dendritic cells [5, 6]. Mono-colonization with SFB can restore immune deficits of germ-free mice, including induction of germinal center activation in Peyer’s patches, production of immunoglobulin A, and T cell expansion [7]. SFB induce genes associated with inflammation and antimicrobial defenses and increase resistance to the intestinal bacterial pathogen *Citrobacter rodentium* [5]. However, SFB also promote extra-intestinal Th17 responses during autoimmune disease, including autoimmune arthritis in the K/BxN mouse model [8] and experimental autoimmune encephalomyelitis (EAE), a murine model of multiple sclerosis [9]. In addition, non-alcoholic fatty liver disease (NAFLD), a common inflammation-driven sequela of obesity, can be exacerbated or prevented in mice by colonization or antibiotic depletion of SFB, respectively [10].

There has been growing interest in understanding the multilayered crosstalk and interactions between nematodes, commensal bacteria, and the host immune system given differences in disease expression in human populations where enteric helminth parasite infection is controlled compared to where it persists [11]. Nematode infection induces polarized type 2 immunity characterized by increased expression of cytokines such as IL-4, IL-5, IL-13, and IL-25 [12]. Epithelial-derived IL-25 is believed to be an initiating factor for the immune cascade, which stimulates type 2 innate lymphoid cells (ILC2) to release IL-5 and IL-13. Host defense against nematode infection relies on Th2 cytokines IL-4 and IL-13 activating STAT6 signaling pathways, which then leads to up-regulation of various downstream effector molecules as well as stereotypic alterations in gut function. Parasite nematodes also have the capacity to suppress Th1 or Th17 immune responses that influence susceptibility to microbial pathogens as well as the process of autoimmunity. For example, concurrent infection with the parasitic nematode *Heligmosomoides polygyrus bakeri* increased the susceptibility of mice to *C. rodentium* [13]. On the other hand, helminth infection can prevent type 1 diabetes, EAE, Graves’ disease, collagen-induced arthritis, and inflammatory bowel disease (IBD) [14], protect against allergies [15, 16], and improve symptoms in IBD [17, 18], all of which are Th1/Th17-associated inflammatory or autoimmune diseases. So far, the cellular and molecular mechanisms underlying the potent immune modulating activities of nematodes remain elusive.

We sought to investigate the effects of infection with the parasitic model nematode *Nippostrongylus brasiliensis* on the composition of the gut microbiota focusing on segmented filamentous bacteria (SFB), a specific Th17-eliciting commensal bacterium, with the goal to gain mechanistic insight into the immune modulating role of parasitic nematodes. Our data showed, for the first time, that the host type 2 response to parasitic nematode infection can inhibit intestinal SFB and decrease the expression of IL-17-associated genes, possible via modulation of antimicrobial peptide and mucin expression.

## Results

### *Nippostrongylus brasiliensis* infection induces type 2 immunity and changes the expression of antimicrobial peptides and mucins in the small intestine

**Fig. 1:**
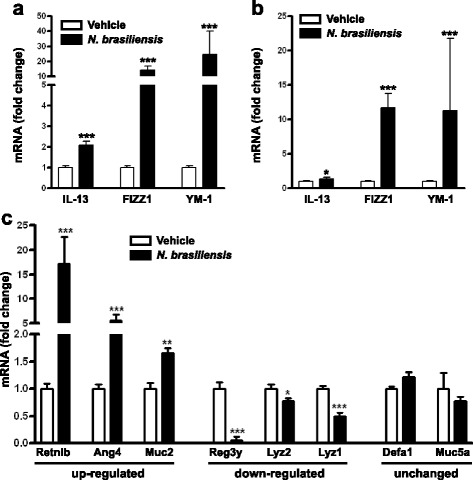
Infection with *N. brasiliensis* induces characteristic alterations in intestinal expression of type 2 cytokines, markers for M2 macrophages, and antimicrobial peptides and mucins. *N. brasiliensis*-infected mice were euthanized at day 11 post-inoculation with infective larvae. qPCR was carried out to examine gene expression of IL-13, Fizz1, and YM-1 in the jejunum (**a**) and ileum (**b**), as well as selected AMP and mucins in the ileum (**c**). The fold change is relative to vehicle after normalization to 18S rRNA. Data shown in *bar graphs* are the mean ± s.e.m. Two-tailed Student’s *t*-test was used for comparisons between infected and uninfected mice. *P < 0.05, **P < 0.01, ***P < 0.001 versus respective vehicle (*n* = 10 for vehicle group and *n* = 9 for *N. brasiliensis*-infected group). *Ang4* angiogenin 4, *Defa1* defensin alpha 1, *Fizz1* found in inflammatory zone 1, *Lyz1/2* lysozymes 1/2, *Muc2* mucin 2, *Muc5Ac* mucin 5 AC, *Reg3γ* regenerating islet-derived protein 3 gamma, *Retnlb* resistin-like molecule beta, YM-1

Enteric nematode infection induces a polarized T-helper 2 (Th2) immune response pivotal to host defense against the infection [12]. *Nippostrongylus brasiliensis* is a rodent gastrointestinal nematode that preferentially colonizes the proximal small intestine after migration through the skin and lung. The infection is acute in immune competent mice with clearance of adult worms from the intestine by day 9–10 after inoculation of infective third-stage larvae (L3) into the skin [19]. Expression of IL-13 and markers for alternatively activated macrophages (M2) (FIZZ1 and YM-1) are up-regulated in both the jejunum and ileum of mice infected with *N. brasiliensis* (Fig. 1a and 1b, respectively).

To determine whether *N. brasiliensis* infection altered the gene expression of antimicrobial peptides (AMP) and mucins in the ileum, we analyzed samples from mice collected at day 11 post-inoculation. The infection up-regulated the expression of resistin-like molecule beta (Retnlb), angiogenin 4 (Ang4), and mucin 2 (Muc2) but down-regulated regenerating islet-derived protein 3 gamma (Reg3γ) and lysozymes 1 (Lyz1) and 2 (Lyz2) (Fig. 1c). Expression of mucin 5 AC (Muc5AC), a pivotal player for worm expulsion [20], or defensin alpha 1 (Defa1) was not affected by the infection at this time (Fig. 1c).

### Infection with *N. brasiliensis* reduces Firmicutes but increases Bacteroidetes and Actinobacteria in the ileum

**Fig. 2:**
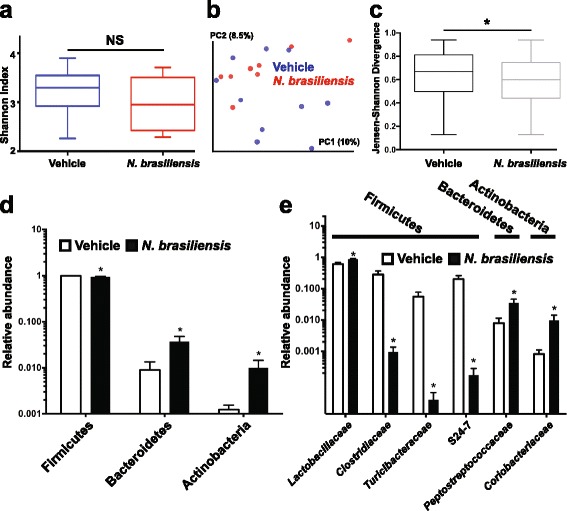
Infection with *N. brasiliensis* modulates the ileal microbiota in mice. Mice infected with *N. brasiliensis* were euthanized at day 11 post-inoculation and metagenomic DNA isolated from ileal tissue samples including luminal content and subjected to 16S rRNA gene short amplicon sequencing. **a** Microbial diversity calculated with the Shannon diversity index and compared with the Mann–Whitney test. **b** Qualitative (un-weighted UniFrac) and **c** quantitative (weighted UniFrac) phylogenetic distance calculations. **d** Taxonomic phyla and **e** families with differential abundance in *N. brasiliensis*-infected compared to uninfected mice as calculated with the non-parametric Fisher test as implemented in Metastats. Data shown in *bar graphs* are the mean ± s.e.m. *P < 0.05 versus vehicle (*n* = 10 for vehicle group and *n* = 9 for *N. brasiliensis*-infected group)

Infection of mice with the parasitic nematode *H. polygyrus bakeri* was shown to increase the total bacterial load and specifically the abundance of *Lactobacillaceae* in the ileum [21]. To determine whether *N. brasiliensis* also induced changes in the intestinal microbiota, 16S rRNA gene amplicon sequencing was performed on metagenomic DNA isolated from ileal samples collected at day 11 post-inoculation. No significant difference in alpha diversity, i.e., species richness and evenness (Shannon diversity), was identified between infected and uninfected mice (Fig. 2a). However, a comparison of ileal microbiota composition between *N. brasiliensis*-infected and uninfected mice showed significantly more clustering in samples from the ileum of infected mice (*p* < 0.05, Jensen-Shannon divergence) (Fig. 2b, c). These results suggest that *N. brasiliensis* infection reduced inter-individual taxonomic microbiota variation.

Infection with *N. brasiliensis* also correlated with significant changes in the abundance of the three dominant bacterial phyla Firmicutes (98.7 ± 0.6 % in uninfected versus 94.7 ± 1.6 % in infected mice) Bacteroidetes (0.9 ± 0.4 % in uninfected versus 3.7 ± 1.1 % in infected mice) and Actinobacteria (0.1 ± 0.03 % in uninfected versus 1 ± 0.4 % in infected mice) (Fig. 2d). Among the Firmicutes, *N. brasiliensis* infection significantly increased the abundance of *Lactobacillaceae* but decreased the abundance of *Peptostreptococcaceae*, *Clostridiaceae*, and *Turicibacteraceae* (Fig. 2e). Members of the taxonomic families S24-7 and *Coriobacteriaceae* of the Bacteroidetes and Actinobacteria, respectively, were significantly increased in *N. brasiliensis*-infected compared to uninfected mice.

### Abundance of SFB and expression of IL-17-associated genes are reduced in *N. brasiliensis*-infected mice

**Fig. 3:**
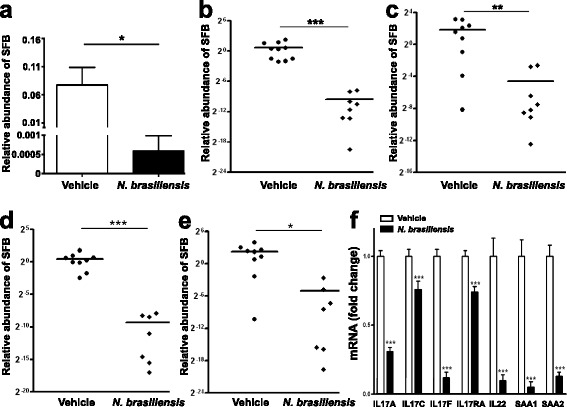
Infection with *N. brasiliensis* reduces the abundance of segmented filamentous bacteria (SFB) along the gastrointestinal tract and the expression of Th17 cell-associated genes in the ileum. Metagenomic DNA was extracted from intestinal strips containing the luminal contents or feces. 16S rRNA sequencing was carried out to examine the relative abundance of SFB in the ileum (**a**). qPCR further confirmed the decrease of SFB abundance in infected mice in ileum (**b**), jejunum (**c**), proximal colon (**d**), and feces (**e**), relative to vehicle after normalization to total bacteria. Expression of Th17-associated genes was determined by qPCR (**f**). The fold change in mRNA is relative to vehicle after normalization to 18S rRNA. Data shown in bar graphs are the mean ± s.e.m. Mann–Whitney test (**a**–**e**) or two-tailed Student’s *t*-test (**f**) was used for comparisons between groups. *P < 0.05, **P < 0.01, and ***P < 0.001 versus respective vehicle (*n* = 10 for vehicle group and *n* = 9 for *N. brasiliensis*-infected group)

SFB are a group of commensal, Gram-positive, anaerobic, and spore-forming bacteria within the phylum Firmicutes with a characteristic long filamentous morphology [3]. They were described and studied mainly in mice where SFB are closely associated with the ileal epithelium [5], but SFB also colonize other vertebrate species including humans [4]. Based on 16S rRNA amplicon sequence data, the relative abundance of SFB (*Candidatus arthromitus*, GenBank accession: CP008713) alone could discriminate between ileal samples from *N. brasiliensis*-infected and uninfected mice (Fig. 3a) accounting for 7.7 ± 3.1 % of all 16S rRNA sequences in ileal samples from the uninfected mice but only 0.06 ± 0.04 % from *N. brasiliensis*-infected mice. This dramatic reduction of SFB in infected mice was confirmed by quantitative real-time PCR (qPCR) showing that the amount of SFB in the ileum was decreased >2000 fold (Fig. 3b). In addition, SFB were more generally reduced in jejunum (Fig. 3c), cecum (not shown), proximal colon (Fig. 3d), and feces (Fig. 3e).

We sought to examine the expression of host IL-17-associated genes in ileal tissue of *N. brasiliensis*-infected mice because helminthic infection has been shown to inhibit pro-inflammatory Th1/Th17 responses [22, 12]. Transcript levels of all tested IL-17-associated genes were significantly lower in ileal tissues from *N. brasiliensis*-infected compared to uninfected mice, including IL-17A, IL-17C, IL-17F, IL-17RA, IL-22, as well as Th17-inducing serum amyloid A proteins SAA1 and SAA2 (Fig. 3f).

### Infection of SFB-negative mice with *N. brasiliensis* does not affect ileal IL-17 expression

**Fig. 4:**
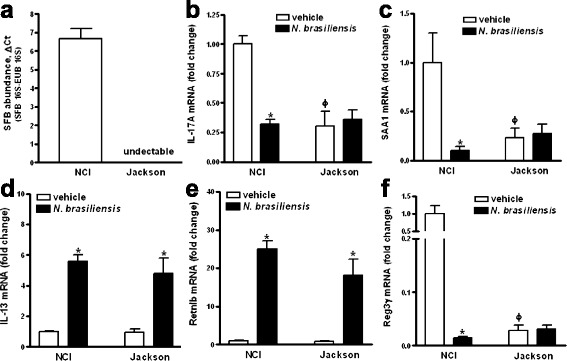
Infection with *N. brasiliensis* does not affect ileal expression of IL-17-associated genes in SFB-negative mice. Mice from NCI-Frederick (NCI) or Jackson Laboratory (Jackson mice) were infected with *N. brasiliensis* and metagenomic DNA extracted from ileal strips containing luminal contents or feces. qPCR was carried out to examine the relative abundance of SFB (**a**). Total RNA was isolated from the ileum and expression of IL-17A (**b**), SAA1 (**c**), IL-13 (**d**), Retnlb (**e**), or Reg3γ (**f**) was determined by qPCR. The fold change in mRNA is relative to NCI vehicle after normalization to 18S rRNA. Data shown in *bar graphs* are the mean ± s.e.m. *P < 0.05 versus respective vehicle; ^ϕ^ *P* < 0.05 versus NCI vehicle (*n* = 6–8 for each group), based on one-way ANOVA followed by Newman-Keuls test

Mice from the Jackson Laboratory (Jackson mice) are not colonized with SFB and provide a useful model to test for SFB-independent host effects [5]. To test whether the reduction of IL-17-associated gene expression in *N. brasiliensis*-infected mice is dependent on SFB, we infected Jackson mice alongside mice from our conventional source of the Frederick National Laboratory for Cancer Research (NCI mice) used in previous experiments. Both 16S rRNA pyrosequencing and qPCR repeatedly showed ileal colonization of NCI mice with SFB but failed to detect SFB in ileal samples from Jackson mice (Fig. 4a and data not shown). Consistent with previous findings [5], basal expression of IL-17-associated genes was significantly lower in Jackson mice compared to NCI mice (Fig. 4b, c and not shown). Importantly, *N. brasiliensis* infection had no significant effect on IL-17-associated ileal gene expression in SFB-negative Jackson mice contrary to that observed in SFB-positive NCI mice (Fig. 4b, c and not shown). However, infection induced the type 2 cytokine IL-13 (Fig. 4d) and the AMP Retnlb (Fig. 4e) and Ang4 (not shown) in both Jackson and NCI mice. In addition, constitutive ileal expression of Reg3γ in uninfected Jackson mice was much lower than in NCI mice and down-regulation of Reg3γ in infected NCI mice was absent in infected Jackson mice (Fig. 4f).

### *N. brasiliensis*-induced modulation of AMP, SFB, and IL-17 depends on host IL-13/STAT6 axis

**Fig. 5:**
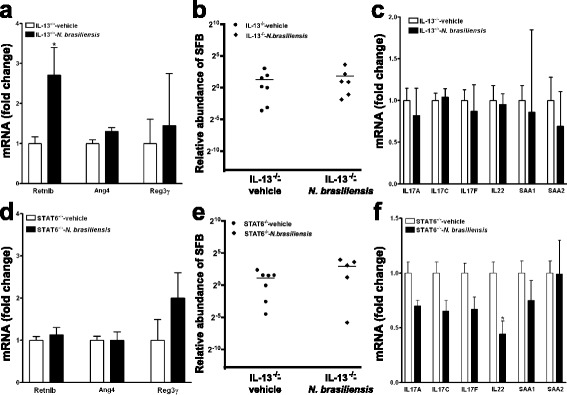
*N. brasiliensis* infection-induced alterations in AMP, SFB, and Th17-associated genes depend primarily on IL-13 and STAT6. Mice deficient in IL-13 (**a**–**c**) or STAT6 (**d**–**f**) were infected with *N. brasiliensis* and euthanized at day 11 post-inoculation. qPCR was carried out to quantify expression of antimicrobial peptides (**a**, **d**), SFB-specific and universal bacterial 16S rRNA (**b**, **e**), and Th17-associated genes (**c**, **f**). The abundance of SFB is relative to vehicle after normalization to total bacteria based on 16S rRNA qPCR. The fold change in mRNA is relative to vehicle after normalization to 18S rRNA. Data shown in *bar graphs* are the mean ± s.e.m. Mann–Whitney test (**b**, **e**) or two-tailed Student’s *t*-test (**a**, **c**, **d**, **f**) was used for comparisons between the two groups. *P < 0.05 versus respective vehicle (*n* = 7 for vehicle groups and 6 for *N. brasiliensis-*infected groups; one mouse died after infection)

The host defense of mice against *N. brasiliensis* infection relies mainly upon activation of the STAT6 signaling pathway by IL-13 [19]. To determine whether the host IL-13/STAT6 axis contributed to infection-induced changes in SFB abundance and associated Th17 responses, mice deficient in IL-13 (IL-13^−/−^) or STAT6 (STAT6^−/−^) were infected with *N. brasiliensis*. While both IL-13^−/−^ and STAT6^−/−^ mice were unable to expel worms, no significant differences in ileal SFB abundance were detected among naïve, uninfected WT, IL-13^−/−^, or STAT6^−/−^ mice (Additional file 1: Figure S1). Infection-induced alterations in ileal AMP expression seen in *N. brasiliensis*-infected WT mice, including up-regulation of Retnlb and Ang4 and down-regulation of Reg3γ, disappeared almost entirely in IL-13^−/−^ or STAT6^−/−^ mice (Fig. 5a, d). Consistent with these results and in contrast to what we observed in WT mice, *N. brasiliensis* infection of IL-13^−/−^ or STAT6^−/−^ mice did not significantly affect ileal SFB concentrations (Fig. 5b, e) nor expression levels of IL-17-associated genes (Fig. 5c, f).

### Exogenous IL-25 modulates AMP, SFB, and IL-17 gene expression similar to infection with *N. brasiliensis*

**Fig. 6:**
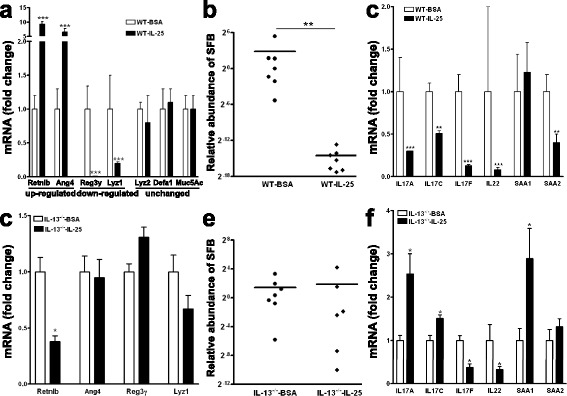
Exogenous IL-25 induces type 2 immunity, as well as IL-13-dependent alterations in ileal AMP expression and SFB abundance. WT (**a**–**c**) or IL-13^−/−^ (**d**–**f**) mice were injected with IL-25 (i.p., 1 μg per mouse) daily for 3 days and euthanized at day 4. qPCR was carried out to examine expression of antimicrobial peptides (**a**, **d**), SFB-specific and universal bacterial 16S rRNA (**b**, **e**), and Th17-associated genes (**c**, **f**) in the ileum. The abundance of SFB is relative to vehicle after normalization to universal bacterial 16 s rRNA. The fold change is relative to vehicle after normalization to 18S rRNA. Data shown in *bar graphs* are the mean ± s.e.m. Mann–Whitney test (**b**, **e**) or two-tailed Student’s *t*-test (**a**, **c**, **d**, **f**) was used for comparisons between the two groups. *P < 0.05, **P < 0.01, ***P < 0.001 versus respective vehicle (*n* = 7 for BSA groups and WT-IL-25, *n* = 6 for IL-13^−/−^-IL-25)

To determine whether exogenous administration of a type 2-promoting cytokine can mimic the effects of *N. brasiliensis* infection, mice were injected daily for 3 days with the recombinant cytokine IL-25. IL-25 promotes type 2 while inhibiting Th1/Th17 immunity [23, 24]. Indeed, exogenous IL-25 significantly increased the expression of IL-13 as well as markers for M2 macrophages, arginase-1 and YM-1, in the ileum (Additional file 2: Figure S2). Exogenous IL-25 also selectively modulated expression of specific AMP and mucins in the ileum, including up-regulation of Retnlb and Ang4, and down-regulation of Reg3γ and Lyz1 (Fig. 6a). Finally, exogenous IL-25 significantly decreased the abundance of SFB (Fig. 6b) and the expression of IL-17-associated genes (Fig. 6c) in the ileum similar to infection with *N. brasiliensis*.

Subsequently, exogenous IL-25 was injected to IL-13^−/−^ mice. In contrast to what was observed in WT mice, administration of IL-25 to IL-13^−/−^ mice did not modulate ileal expression of Ang4, Reg3γ, or Lyz1 but down-regulate Retnlb (Fig. 6d). Consistent with these results, IL-25 had no significant effect on ileal SFB in IL-13^−/−^ mice (Fig. 6e). Although exogenous IL-25 down-regulated the expression of IL-17 F and IL-22 in IL-13^−/−^ mice, albeit to a lesser degree than in WT mice, the inhibitory effects of IL-25 in WT mice on the key IL-17-associated genes IL-17A and IL-17C disappeared in IL-13^−/−^ mice. An up-regulation of IL-17A, IL-17C, and SAA1 was detected in IL-25-treated IL-13^−/−^ mice (Fig. 6f).

## Discussion

The effects of nematode infection on the intestinal microbiota have been studied with differing results in humans. While various types of helminth infection were associated with altered microbiota composition in a Malaysian indigenous community [25], as well as in children from Ecuador colonized with *Trichuris trichiura* and *Ascaris lumbricoides* but not *T. trichiura* alone [26], no measurable effect on the microbiota structure was seen in participants of a clinical study infected with *Necator americanus* [27]. In summary, additional studies using larger, more standardized patient cohorts and localized tissue sampling may be required [28, 29] to identify potential effects of parasite infection on the human intestinal microbiota.

Host-derived mucins and AMP control the composition and spatial organization of the intestinal microbiota and are induced in response to parasite and other enteric infections [30]. Resistin-like molecule β (Retnlb), for example, provides protection against *N. brasiliensis* and *H. polygyrus* in mice, independently of T or B cells or M2 macrophages, by interfering with the parasite’s ability to feed on host tissue during infection [31]. Mucin glycoproteins secreted by goblet cells concentrate AMP produced by Paneth cells and protect the intestinal lining from direct contact with luminal microorganism. Proliferation of intestinal goblet cells is a prominent feature of the type 2 immune response to nematode infection [32, 33] and both Muc2 and Muc5ac are important for worm expulsion [20, 34]. At the same time, mucus-derived glycans are an important energy source for intestinal bacteria [34]. We found the expression of Muc2 and several AMP with broad antimicrobial activity to be altered in response to *N. brasiliensis* infection, including Ang4 (effective against Gram-positive/-negative bacteria), Reg3γ (effective against Gram-positive bacteria), and Lyz1 and Lyz2 (mostly effective against Gram-positive bacteria) [30]. Together, our results suggest that parasite-induced changes to intestinal mucus architecture and AMP expression profiles could be responsible for the altered intestinal microbiota observed in parasite-infected mice.

Notably, our observed increase in *Lactobacillaceae* in the ileum of *N. brasiliensis*-infected mice was consistent with a similar increase found in ileal but not cecal samples of *H. polygyrus*-infected mice [21]. To our knowledge, however, our study is the first to report a reduction in SFB in response to parasite infection, a bacterial species that has received widespread attention due to its unique Th17-inducing and immune modulating capabilities.

Parasite expulsion is associated with smooth muscle hyper-contractility, epithelial cell hypo-secretion, and increased mucosal permeability, mediated primarily via IL-4/IL-13 and receptor-mediated activation of STAT6 signaling pathways [35, 36]. As IL-13^−/−^ mice fail to reduce SFB and IL-17A expression in response to *N. brasiliensis* infection, IL-4 appears unable to compensate for IL-13 with regard to its role for SFB and IL-17-dependent immune modulation. Because SFB colonize the ileal intestinal wall [37], reduced concentrations of SFB in *N. brasiliensis*-infected mice could result from increased physical shedding of the intestinal mucus or from selectively increased AMP expression [38]. Germ-free mice mono-colonized with SFB have been shown to induce Reg3γ [39], suggesting that the reduced Reg3γ expression in mice infected with *N. brasiliensis* was associated with lower SFB colonization or with distinct host cytokine regulatory pathways activated by parasite infection. Reduced levels of Reg3γ could affect the Gram-positive intestinal microbiota and lead to the increase in *Lactobacillaceae* observed in *N. brasiliensis*-infected mice. Mucosal IgA levels could also play a role for maintaining SFB homeostasis, as mice deficient for IgA, as well as mice that lack the TLR adaptor MyD88 in Treg cells, which results in impaired intestinal IgA responses, show an expansion of intestinal SFB [40, 41]. Our findings suggest complex interactions between parasite, host response, and microbiota that require further study.

Helminth infection impairs the human immune response to oral cholerae [42], tuberculosis vaccine BCG [43] and exacerbates *Salmonella enterica* Typhimurium pathogenicity in mice [44]. Moreover, human Th17 responses to latent tuberculosis infection are reduced in individuals colonized with helminths [45, 46], with clinical consequences for large parts of the developing world where *Mycobacterium tuberculosis* and helminth infections are co-endemic [44]. Suppression of Th1 and Th17 responses as well as attenuation of EAE, a murine model of the human autoimmune disease multiple sclerosis, was maintained in IL-10 knock-out mice infected with the liver fluke *Fasciola hepatica* [47]. In *H. polygyrus*-infected mice, IL-17 suppression was not affected by blocking of IL-10 alone but by blocking of both IL-10 and the Th2 cytokine IL-4 [48]. Our findings that parasite-induced IL-17 suppression is dependent on the IL-13/STAT6 axis and inducible solely by administration of IL-25 which also reduced SFB levels support and extend the link between Th2 and Th17 responses during parasite infection and help explain increased comorbidity to bacterial infections in parasite-infested individuals.

Parasite infection and colonization with SFB have been associated with beneficial and detrimental effects as results of opposing immune modulating roles, i.e., increased susceptibility to microbial infection and protection from inflammatory disorders in the case of parasite infection and increased resistance to some microbial infection and susceptibility to inflammatory disease in case of SFB. Our finding that parasite infection reduced the abundance of SFB and IL-17 activation could help explain epidemiological reports of negative correlations between parasite infection and autoimmune disease [11] as well as the success seen in the treatment of some inflammatory and autoimmune diseases with helminth therapy [22].

In light of the pro-inflammatory role of IL-17 for autoimmune disease, IL-17-inducing SFB could represent a valuable therapeutic target. However, only a few factors have been described that control SFB abundance and the mechanisms that are responsible for SFB regulation are largely unknown: Immune system deficiencies including loss of function of the MyD88 adaptor protein [49] used by most Toll-like receptors [49], lymphotoxin (TNFβ) [50] required for normal mucosal immunity [50], and the aryl hydrocarbon receptor [51] involved in the activation of group 3 ILCs [51] have been associated with increased intestinal SFB concentrations in mice. Shi et al. showed that IL-23 dynamically regulated SFB, as perturbation of the IL-23 pathway led to defective intestinal barrier function, systematic dissemination of microbial products, and altered antimicrobial activities [52].

## Conclusions

**Fig. 7:**
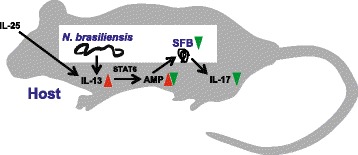
Proposed model for the immune regulatory network that connects host, parasitic nematode, and SFB microbiota during *N. brasiliensis* infection in mice. We showed that SFB-colonized mice reduce SFB and expression of IL-17 upon infection with *N. brasiliensis* or induction with exogenous IL-25 in a way that is dependent on STAT6 activation by IL-13 and possibly modulation of intestinal AMP and mucin expression. This is consistent with a model in which the host type 2 response to parasite infection leads to SFB depletion with the net result of reduced intestinal IL-17 expression

Gastrointestinal immune homeostasis depends on complex interactions between the host and various inhabitants of the gastrointestinal tract, including parasites and microbiota. Here, we show that infection of mice with *N. brasiliensis* induced a type 2 immunity-dependent reduction of intestinal SFB, associated with reduced intestinal IL-17 expression (Fig. 7). Our findings provide for the first time evidence that changes in the intestinal microbiota could be linked to immune modulating effects associated with parasite infection. They further suggest that alterations to the mucosal layer organization and AMP expression profiles mediated by cytokine- and parasite-induced Th2 responses should be studied to identify new therapeutic strategies to manipulate SFB and IL-17 production.

## Methods

### Mice

C57BL/6 WT mice were purchased from NCI-Frederick Animal Production Program (Frederick, MD) and bred in the USDA/Beltsville animal facility. These mice were confirmed to be SFB positive by both 16S pyrosequencing and qPCR. SFB-negative WT mice in C57BL/6 background were purchased from Jackson Laboratory (Bar Harbor, ME). Jackson mice were housed in autoclaved cages in our closed container facility that have a sealed top and force-filtered air, feed autoclaved chow and water, handled in a biological safety hood with HEPA-filtered air, and used 1 week after arriving. Mice deficient in STAT6 (STAT6^−/−^) on C57BL/6 background from Jackson laboratory and mice deficient in IL-13 (IL-13^−/−^) from the NIAID Taconic contract were bred in the USDA/Beltsville animal facility. Both STAT6^−/−^ and IL-13^−/−^ mice were confirmed to be SFB positive before use. In general, mice were co-housed for 1 week before initiating treatment/infection to eliminate cage effects. Unless otherwise indicated, 8–12-week-old female mice were used throughout the study with 5–10 per group based on our previous studies using *N. brasiliensis* infection [53]. Mice were randomly assigned to different treatment groups. Investigators were aware of the group allocation throughout the experiment. These studies were conducted with institutional approval from both the University of Maryland, Baltimore and the USDA Beltsville Area Institutional Animal Care and Use Committees, in accordance with principles set forth in the Guide for Care and Use of Laboratory Animals, Institute of Laboratory Animal Resources, National Research Council, Health and Human Services Publication (National Institutes of Health 85–23, revised 1996).

### Infection of mice with *N. brasiliensis*

Infective, third-stage larvae of *N. brasiliensis* (specimens on file at the U.S. National Parasite Collection, U.S. National Helminthological Collection, Collection 81930, Beltsville, MD) were propagated and stored at room temperature in fecal/charcoal/peat moss culture plates until used. Groups of mice were inoculated subcutaneously with 500 third-stage larvae (L3) and euthanized at day 11 post-*N. brasiliensis* infection when immunocompetent mice cleared worms, as described previously [36]. Appropriate age- and sex-matched WT or mice treated with vehicle were performed for each infection.

### Administration of IL-25

For administration of IL-25, mice were injected i.p. with 1 μg of mouse recombinant IL-25, containing BSA as a carrier (Biolegend, CA) in 100-μl saline daily for 3 days or, as a control, injected with 35-μg BSA, which is equal to the amount of BSA included in the IL-25 preparation. The amount of cytokine administered was based on the effective dose of IL-25 that induced a prominent Th2 immune response from a previous study^51^.

### Nucleic acid extraction and qPCRs

Intestinal strips containing the entire luminal contents were placed in RNAlater (Sigma-Aldrich, St. Louis, MO) and stored at −80 °C until processing. Prior to processing, samples were vortexed vigorously for 15 s and used for total DNA (supernatant) or RNA (tissue) isolation. Total RNA was extracted from mid-jejunum or ileum whole tissue and used for cDNA generation and real-time quantitative PCR (qPCR) as described previously [54]. RNA samples (2 μg) were reverse transcribed to cDNA using the First Strand cDNA Synthase Kit (MBI Fermentas, Hanover, MD) with random hexamer primer. Real-time qPCR (qPCR) was performed on a CFX96 detection system (Bio-Rad, Hercules, CA). PCR was performed in a 25-μl volume using SYBR Green Supermix (Bio-Rad). Amplification conditions were: 95 °C for 3 min, 50 cycles of 95 °C for 15 s, 60 °C for 15 s, and 72 °C for 20 s. The fold changes in mRNA expressions for targeted genes were relative to the respective vehicle groups of mice after normalization to 18S rRNA. Primers were synthesized by Sigma-Aldrich. Results are presented as mean values and standard error of the mean (mean ± s.e.m.). Metagenomic DNA was isolated from mouse intestine or fecal pellets using the protocol for human fecal DNA isolation described in Song et al. [55], which includes both enzymatic digestions (lysozyme, mutanolysin, lysostaphin, proteinase K, and RNase) and mechanical disruption by bead beating. Segmented filamentous bacteria were quantified by qPCR using primer pairs specific for the SFB 16S rRNA gene or, for normalization, 16S rRNA genes from all bacteria as described in Barman et al. [56]. Fifty nanograms of DNA template was amplified using the SYBR Green PCR Master Mix (Life Technologies, Carlsbad, CA) in a 10-μl reaction mix following the default amplification protocol of the ABI 7900HT Real-Time PCR system (Life Technologies). For SFB, relative quantity was calculated by the ΔCt method and normalized by counts for total bacteria. Typical Ct values for SFB were ~30 cycles and for total bacteria ~15 cycles. Concentrations for samples that were negative after 40 cycles were designated “not detectable” (n.d.).

### 16S rRNA gene amplification, sequencing, and microbiota analysis

Barcoded 16S rRNA gene PCR amplicons for sequencing were generated a described previously [57]. Hypervariable regions V3 and V4 of the bacterial 16S rRNA gene were amplified with the universal primers 338F and 806R (amplicon length: ~470 bp). Reads spanning hypervariable region V3 were sequenced with primer 338 on the Illumina MiSeq platform. Quality trimming was performed as described before [55], using the following criteria: (1) reads were truncated upstream of >2 consecutive low-quality bases; (2) no reads with ambiguous base calls were used; and (3) reads with <150 bp after trimming were discarded. Quality trimming and demultiplexing were performed with QIIME (version 1.6.0) [58], resulting in between 3058 and 17,643 reads per sample. Sequences were clustered as operational taxonomic units (OTUs) based on a 97 % cutoff with USEARCH, *de-novo* chimera detection and removal were conducted with UCHIME v5.1 as implemented in QIIME. Taxonomic ranks were assigned to each sequence with the Ribosomal Database Project (RDP) Naïve Bayesian Classifier v.2.2, using a pre-built Greengenes database of 16S rRNA sequences [May, 2013] and a confidence value cutoff of 0.9, also as implemented in QIIME. Differentially abundant OTUs were determined with Metastats [59]. Raw sequences of all non-chimeric reads that passed the quality trimming were deposited in the NCBI Short Read Archive under accession numbers SRA176950 and SRP045195 (Bioproject ID: PRJNA255974).

### Statistical analyses

The Shapiro-Wilk test was used to evaluate the normality of the obtained data. Outliers were identified using the ROUT method and excluded from the analysis. If necessary, variance between compared groups was corrected with the Geisser-Greenhouse method. One-way ANOVA followed by Newman-Keuls test was performed for comparisons of more than two groups. When normal distribution was satisfied, Student’s *t-*test was used for comparing differences between groups. Otherwise, the non-parametric Mann–Whitney test was used. All analyses and plots were conducted using Prism (version 6 for Mac, GraphPad Software, San Diego, CA, USA). Results are presented as mean ± s.e.m. Statistical significance was declared if the two-sided *P* value was <0.05.

## Additional files

Additional file 1: Figure S1. Deficiency in IL-13 or STAT6 is not associated with altered intestinal abundance of segmented filamentous bacteria (SFB). Metagenomic DNA was extracted from ileal strips of wild type (WT), IL-13/- and STAT6-/- mice. qPCR was carried out to quantify SFB-specific and universal bacterial 16S rRNA. Relative quantities were calculated by the ΔCt method and normalized by counts for total bacteria. The fold change in abundance is relative to wild type mice. (PDF 354 kb) Additional file 2: Figure S2. Exogenous administration of IL-25 induces intestinal expression of type 2 cytokines (IL-13) and markers for M2 macrophages (Arginase-1, YM-1). qPCR was carried out to examine gene expression of IL-13, Arginase-1, and YM-1 in the ileum of wild type mice treated with IL-25 or BSA. The fold change is relative to vehicle after normalization to 18S rRNA. Data shown in bar graphs are the mean ± s.e.m. Two-tailed Student’s t-test was used for comparisons between the two groups of mice. ***P < 0.001 versus respective vehicle (n = 10 for both groups). (PDF 387 kb)
