# Erratum to: Type 2 immunity-dependent reduction of segmented filamentous bacteria in mice infected with the helminthic parasite *Nippostrongylus brasiliensis*

**DOI:** 10.1186/s40168-015-0142-1

**Published:** 2015-12-21

**Authors:** W. Florian Fricke, Yang Song, An-Jiang Wang, Allen Smith, Viktoriya Grinchuk, Chenlin Pei, Bing Ma, Nonghua Lu, Joseph F. Urban, Terez Shea-Donohue, Aiping Zhao

**Affiliations:** Department of Microbiology and Immunology, Institute for Genome Sciences, University of Maryland School of Medicine, Baltimore, MD USA; Department of Nutrigenomics, University of Hohenheim, Stuttgart, Germany; Department of Radiation Oncology, University of Maryland School of Medicine, Baltimore, MD USA; Department of Gastroenterology and Hepatology, The First Affiliated Hospital of Nanchang University, Nanchang, China; U.S. Department of Agriculture, Agriculture Research Service, Beltsville Human Nutrition Research Center, Diet, Genomics, and Immunology Laboratory, Beltsville, MD USA

In our original published manuscript entitled “Type 2 immunity-dependent reduction of segmented filamentous bacteria in mice infected with the helminthic parasite *Nippostrongylus brasiliensis*” [1], a co-author was incorrectly omitted.

The correct author list should include Emmanuel Mongodin from the Department of Microbiology and Immunology, Institute for Genome Sciences, University of Maryland School of Medicine, Baltimore, MD, USA, and read “W. Florian Fricke, Yang Song, An-Jiang Wang, Allen Smith, Viktoriya Grinchuk, Emmanuel Mongodin, Chenlin Pei, Bing Ma, Nonghua Lu, Joseph F. Urban, Terez Shea-Donohue and Aiping Zhao”. Correspondingly, the authors contribution should read “WFF and AZ conceived the study concept and design. YS, AJW, VG, EM, CP, and NL did the acquisition of data. WFF, YS, BM, and AZ interpreted and analyzed the data. WFF, YS, and AZ drafted the manuscript. WFF, JFU, TSD, and AZ made the critical revision of the manuscript. YS and AZ did the statistical analysis. WFF and AZ obtained funding. All authors read and approved the final manuscript”.
