# Supplementary figures and images for: Type 2 immunity-dependent reduction of segmented filamentous bacteria in mice infected with the helminthic parasite Nippostrongylus brasiliensis
Source: Microbiome. 2015 Sep 17;3:40. doi: 10.1186/s40168-015-0103-8 (PMC4574229; doi:10.1186/s40168-015-0103-8)

Fig. S1

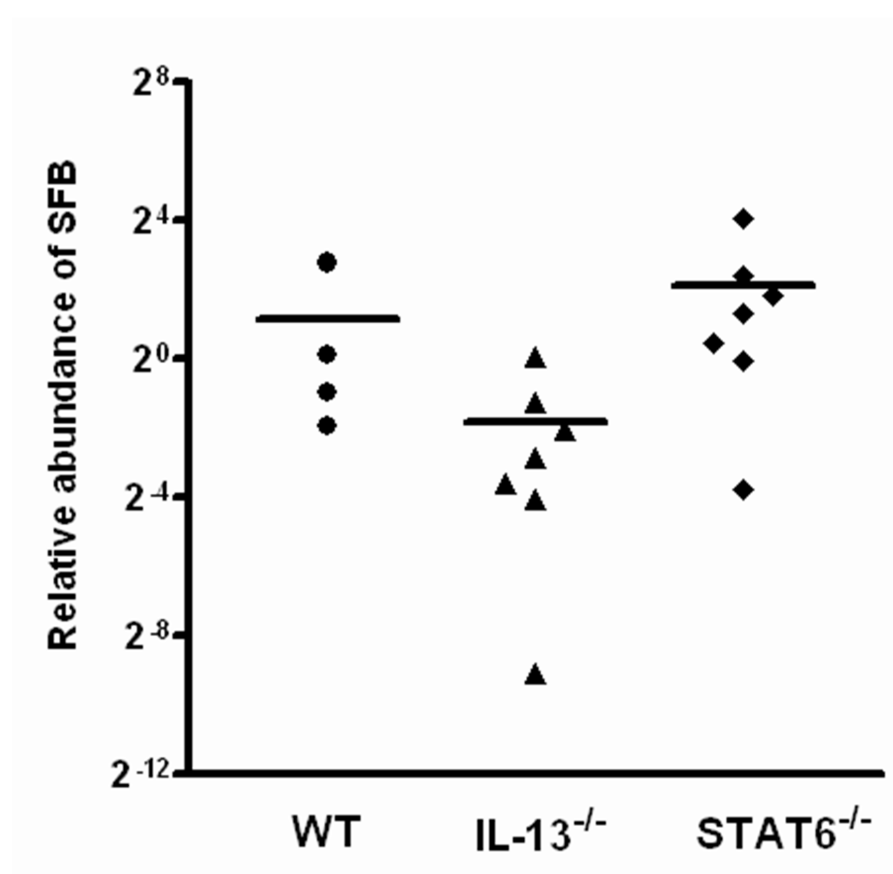

Supplement: Additional file 1: Figure S1. — Deficiency in IL-13 or STAT6 is not associated with altered intestinal abundance of segmented filamentous bacteria (SFB). Metagenomic DNA was extracted from ileal strips of wild type (WT), IL-13/- and STAT6-/- mice. qPCR was carried out to quantify SFB-specific and universal bacterial 16S rRNA. Relative quantities were calculated by the ΔCt method and normalized by counts for total bacteria. The fold change in abundance is relative to wild type mice. (PDF 354 kb) [file 40168_2015_103_MOESM1_ESM.pdf]

Fig. S2

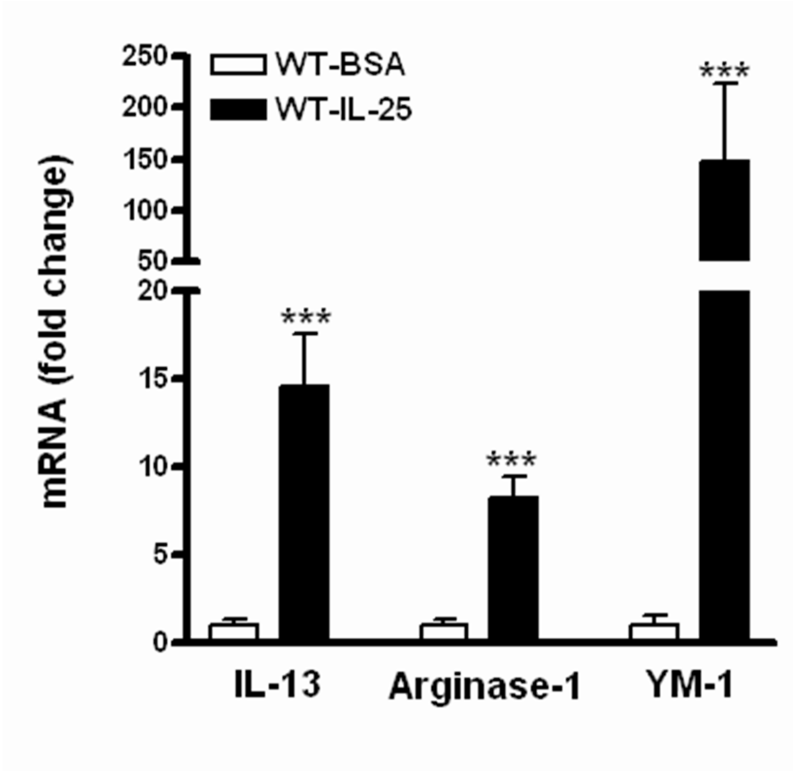

Supplement: Additional file 2: Figure S2. — Exogenous administration of IL-25 induces intestinal expression of type 2 cytokines (IL-13) and markers for M2 macrophages (Arginase-1, YM-1). qPCR was carried out to examine gene expression of IL-13, Arginase-1, and YM-1 in the ileum of wild type mice treated with IL-25 or BSA. The fold change is relative to vehicle after normalization to 18S rRNA. Data shown in bar graphs are the mean ± s.e.m. Two-tailed Student’s t-test was used for comparisons between the two groups of mice. ***P < 0.001 versus respective vehicle (n = 10 for both groups). (PDF 387 kb) [file 40168_2015_103_MOESM2_ESM.pdf]
